# Computational Screening of Anti-Cancer Drugs Identifies a New BRCA Independent Gene Expression Signature to Predict Breast Cancer Sensitivity to Cisplatin

**DOI:** 10.3390/cancers14102404

**Published:** 2022-05-13

**Authors:** Jean Berthelet, Momeneh Foroutan, Dharmesh D. Bhuva, Holly J. Whitfield, Farrah El-Saafin, Joseph Cursons, Antonin Serrano, Michal Merdas, Elgene Lim, Emmanuelle Charafe-Jauffret, Christophe Ginestier, Matthias Ernst, Frédéric Hollande, Robin L. Anderson, Bhupinder Pal, Belinda Yeo, Melissa J. Davis, Delphine Merino

**Affiliations:** 1 Olivia Newton-John Cancer Research Institute, Melbourne, VIC 3084, Australia; jean.berthelet@onjcri.org.au (J.B.); farrah.el-saafin@onjcri.org.au (F.E.-S.); antonin.serrano@onjcri.org.au (A.S.); michal.merdas@gmail.com (M.M.); matthias.ernst@onjcri.org.au (M.E.); robin.anderson@onjcri.org.au (R.L.A.); bhupinder.pal@onjcri.org.au (B.P.); belinda.yeo@onjcri.org.au (B.Y.); 2 School of Cancer Medicine, La Trobe University, Bundoora, VIC 3086, Australia; 3 Bioinformatics Division, The Walter and Eliza Hall Institute of Medical Research, Parkville, VIC 3052, Australia; momeneh.foroutan@monash.edu (M.F.); bhuva.d@wehi.edu.au (D.D.B.); whitfield.h@wehi.edu.au (H.J.W.); joseph.cursons@monash.edu (J.C.); 4 Victorian Comprehensive Cancer Centre, The University of Melbourne Centre for Cancer Research, Melbourne, VIC 3000, Australia; frederic.hollande@unimelb.edu.au; 5 Department of Clinical Pathology, The University of Melbourne, Parkville, VIC 3052, Australia; 6 Department of Medical Biology, Faculty of Medicine, Dentistry and Health Science, The University of Melbourne, Melbourne, VIC 3010, Australia; 7 Immunology Division, The Walter and Eliza Hall Institute of Medical Research, Parkville, VIC 3052, Australia; 8 Department of Medicine, Faculty of Medicine, Dentistry and Health Science, The University of Melbourne, Melbourne, VIC 3010, Australia; 9 Garvan Institute of Medical Research, Darlinghurst, NSW 2010, Australia; e.lim@garvan.org.au; 10 St Vincent’s Clinical School, Faculty of Medicine, UNSW Sydney, Darlinghurst, NSW 2010, Australia; 11 St Vincent’s Hospital, Darlinghurst, NSW 2010, Australia; 12 CRCM, Inserm, CNRS, Institut Paoli-Calmettes, Aix-Marseille, Epithelial Stem Laboratory, Equipe Labellisée LIGUE Contre le Cancer, 13009 Marseille, France; jauffrete@ipc.unicancer.fr (E.C.-J.); christophe.ginestier@inserm.fr (C.G.); 13 Department of Medical Oncology, Austin Health, Melbourne, VIC 3084, Australia; 14 Department of Biochemistry and Molecular Biology, Faculty of Medicine, Dentistry and Health Science, University of Melbourne, Melbourne, VIC 3010, Australia

**Keywords:** breast cancer, pharmacogenomics, predictive modeling, drug sensitivity, precision medicine, cisplatin

## Abstract

**Simple Summary:**

Using a collection of publicly available drug screening resources, we identified different partners of genes associated with either sensitivity or resistance to 90 anti-cancer therapies. When subsequently applying these signatures to multiple datasets, we found that these predictive models could predict a large range of drug responses in patient samples. In particular, we discovered a new gene signature to identify breast cancer tumors that are likely to respond to cisplatin in the absence of BRCA1 mutations. This work constitutes an important advance to accelerate the application of platinum-based therapies in patient groups that are not routinely treated with these drugs. In the future, this approach may help to guide the choice of drugs based on the molecular profile of the tumors.

**Abstract:**

The development of therapies that target specific disease subtypes has dramatically improved outcomes for patients with breast cancer. However, survival gains have not been uniform across patients, even within a given molecular subtype. Large collections of publicly available drug screening data matched with transcriptomic measurements have facilitated the development of computational models that predict response to therapy. Here, we generated a series of predictive gene signatures to estimate the sensitivity of breast cancer samples to 90 drugs, comprising FDA-approved drugs or compounds in early development. To achieve this, we used a cell line-based drug screen with matched transcriptomic data to derive in silico models that we validated in large independent datasets obtained from cell lines and patient-derived xenograft (PDX) models. Robust computational signatures were obtained for 28 drugs and used to predict drug efficacy in a set of PDX models. We found that our signature for cisplatin can be used to identify tumors that are likely to respond to this drug, even in absence of the BRCA-1 mutation routinely used to select patients for platinum-based therapies. This clinically relevant observation was confirmed in multiple PDXs. Our study foreshadows an effective delivery approach for precision medicine.

## 1. Introduction

Breast cancer is a heterogeneous disease with several clinical and molecular subtypes, defined by distinct immunohistochemical, histopathological, and molecular classifications [1,2,3,4]. Measurement of gene expression has long been recognized as a reliable and robust way to assess molecular phenotypes in cancer [2]. Patterns of gene expression (or gene expression signatures) have shown a strong association with clinically meaningful outcomes, such as metastasis and overall survival. Specifically, the classification of molecular subtypes in breast cancer (luminal A/B, triple-negative breast cancer or TNBC, and HER2 amplified breast cancer) based on gene and protein expression has provided a level of refinement in patient stratification and therapeutic decision making. These subtypes have been shown to differ in incidence [5], survival [6,7], and response to therapy [3,8] and they are used to stratify patients for treatment [8,9]. Indeed, patients with luminal A/B or HER2 amplified breast cancer are likely to benefit from endocrine therapy or HER2 targeted therapies, respectively, while TNBC patients are commonly treated with chemotherapy and radiotherapy [10]. However, breast cancer patients within a given subtype often show non-uniform clinical outcomes [11,12], highlighting a need to predict drug sensitivity based on the characteristics of individual tumors.

The identification of clinically relevant driver mutations such as BRCA1, BRCA2, PIK3CA, PTEN, and AKT1, can also be used in the clinic to guide therapeutic decisions [13,14]. For instance, tumors with BRCA-1 mutations are known to be more responsive to PARP inhibitors or platinum-based therapy compared to others [10,15]. However, the number of actionable mutations identified to date remains limited, and their identification is insufficient to accurately predict drug efficacy at the individual level. In this context, the analysis of transcriptomic [16], epigenetic [16,17], proteomic [18], and metabolomic [19] datasets is likely to provide complementary information in the ongoing refinement of precision oncology. 

Different computational approaches have been used on data sets from cancer cohorts to predict drug response in a variety of cancer types [20,21,22], and gene expression data is considered to provide the most useful molecular insight for predicting therapy response in breast cancer [20,23,24]. Gene expression signatures associated with drug efficacy are typically derived from differential expression analysis between drug-sensitive and resistant cell lines [25,26,27]. A comparative analysis of methods for predicting drug response also found that simple, correlation-based methods were surprisingly effective, with performance similar to that of more complicated, data-intensive methods [24]. 

In this study, we used gene expression data from the RNA sequencing of breast cancer cell lines in combination with associated drug response profiles to generate a resampling- and correlation-based computational pipeline to derive gene expression signatures associated with drug efficacy. We then used singscore [28], a single-sample scoring method we developed previously, to generate a drug efficacy score for each cancer cell line using drug-specific gene expression signatures. These scores were then used to build prediction models for 90 drugs and assess their performance by computational validation across several independent datasets. Finally, we validated our predictions using PDX models, focusing on our cisplatin signature, enabling us to identify tumors that are responding to cisplatin despite the absence of a BRCA-1 mutation.

## 2. Materials and Methods

### 2.1. Datasets

All data are listed in Table 1. Pharmacogenomic resources from the Gray lab [20] include transcriptomic data for a large number of breast cancer cell lines with matched drug response data across multiple replicates, and thus, these data were used to fit predicted efficacy signatures for the 90 drugs available. The resulting drug efficacy signatures and prediction models were tested on independent data. 

Cell line pharmacogenomic data were downloaded as PharmacoSets (PSets) through the PharmacoGx R/Bioconductor package (v1.6.1), including: CCLE [4], Cancer Therapy Response Portal (CTRPv2) [30], Genomics of Drug Sensitivity in Cancer (GDSC1000) [29], Genentech Cell Line Screening Initiative (gCSI) [31,32], and Institute for Molecular Medicine Finland (FIMM) [33] data. The GRAY PSet (containing the Gray pharmacogenomic data) was received from the Haibe-Kains’ group in September 2017 and modified (see below). Finally, patient-derived tumor xenografts along with PDTX-derived tumor cells (PDTX-PDTC) from Bruna et al. [34] and different patient cohorts were also examined (Table 1). 

Using data from the PharmacoGX package, we used metrics based on the area above the dose-response curve (activity area; “recomputed AUC” within PSets, and refer to this as AUC in this paper) rather than IC50 values because activity area (or AUC) captures both the efficacy and potency of a drug and further, it is comparable across different cell lines treated by the same drugs and same drug concentrations [38]. 

### 2.2. Deriving Drug Efficacy Signatures

The GRAY PSet was received from the Haibe-Kains’ group (developers of the PharmacoGX package). We re-analyzed the FASTQ files from the Gray lab using the R/Bioconductor packages Rsubread and human genome hg19. Read counts were calculated using featureCount, and the edgeR package [39,40] was used to filter genes (retained genes with a count-per million (CPM) > 2 in at least 10% of cell lines) and calculate log(RPKM) values. For samples with technical replicates, their median log(RPKM) values were calculated and used. These data, as well as the previous microarray data from the Gray lab [9], were appended to the GRAY PSet. 

The log(RPKM) RNA-seq data and “recomputed AUC” drug sensitivity values for all 90 drugs [20] were used in the following analysis. To obtain a drug efficacy signature for each drug, a resampling procedure was used whereby 80% of cell lines were randomly selected (1000 times) and the Spearman’s correlation was calculated between the gene transcript abundance and the drug response metric. Genes found in the top or bottom 3% of correlations across more than 90% of re-sampling runs (i.e., 900 out of 1000) were selected for each drug efficacy signature and are listed in Table S1.

### 2.3. Scoring Samples Using the Singscore and Stingscore Methods

For both training and testing purposes, the singscore (v1.16.0) R/Bioconductor package [28,41,42] was used to score samples using the derived drug efficacy signatures. Genes in drug signatures with positive correlation coefficients were used as the up-regulated gene set, while those with negative correlation coefficients were considered as the down-regulated gene set. Scores obtained from singscore using drug efficacy signatures are called “drug efficacy signature scores” and were used as input to develop prediction models (see next section). PDX samples used for validation purposes were scored using the stingscore methods that use stably expressed genes as anchors to compute scores. The top five stably expressed genes identified in [42] were used to compute drug response scores given a drug response gene expression signature. Scores using stably expressed genes worked better when comparing scores computed across independent datasets [42].

### 2.4. Training Prediction Models

We used five methods to build prediction models with the training data: linear regression, quadratic regression, and three SVM-based models. SVM is a popular supervised method that can be used for classification or regression depending on whether the output is a categorical or continuous variable [43]. In this study, we used linear, polynomial, and radial kernel SVM, in addition to linear and quadratic regression, to examine the performance of a range of linear and non-linear methods in predicting drug response based on the gene expression signatures we derived. First, we converted the gene expression data for each cell line into a score that captured the concordance of the gene expression profile with our drug response expression signature. We used these scores as input features to the various learning methods (SVM and regression) and used the drug response measurement, as captured by the area under the dose-response curve, as the prediction target. We predicted continuous outcomes (in this case, drug response) in the test data sets.

To select model parameters, three-fold cross-validation was performed 20 times for each parameter set. Briefly, data (cell lines) were partitioned into three subsets of similar size, with two used to develop the models while the third was retained for testing. This was repeated three times such that each subset was used once as the test set. This three-fold cross-validation was then repeated 20 times for each drug and each prediction model. To quantify model fit, the RMSE (root mean squared error), MAE (mean absolute error), and R^2^ (R-squared) were calculated for all five methods. Model training and cross-validation were performed using R packages caret and e1071. For each of the five model types, parameters for the final models were selected as those with the best fit in cross-validation across the entire training set. 

Next, we compared the performance of all five methods by calculating the concordance index (CI) and RMSE. The CI quantifies concordance between two ranked vectors (here, predicted and actual drug response) by comparing all pairwise ranks between them [24]. We further calculated the BIC (Bayesian information criterion) to compare regression-based methods (Figure S3). 

### 2.5. Test Prediction Models

Prediction models trained on the Gray data were tested against breast cancer data from the CCLE, GDSC1000, CTRPv2, gCSI, and FIMM panels, as well as the Caldas PDXT-PDTC data [34] (Table 1). Comparisons were performed for all drugs common to the training (Gray data) and test data (CCLE: 11 drugs; CTRPv2: 28; GDSC1000: 29, gCSI: 11; FIMM: 18, and; Caldas: 20 drugs). We tested all models once on the full cell line data, and once after removing the overlapping cell lines between the training and test sets (to examine potential over-fitting). Drug efficacy signatures were also used to score patient data, including samples from TCGA and PDXs (see below) (see Table 1 and Table 2). 

Drugs were classified into high, medium, and low confidence based on the predictive power of their models across multiple independent datasets. As noted above, Spearman’s correlation (*ρ*) and CI were used to examine the strength of association between predicted sensitivity (equivalent to signature scores from linear regression models) and observed drug efficacy. Dependent upon the number of independent data sets available for each drug, we classified: (1) high confidence drugs with *ρ* ≥ 0.4 or CI ≥ 0.65 in at least one test set, (2) low confidence drugs with *ρ* < 0.3 or CI < 0.6 in all available test sets, and (3) medium confidence drugs including those that do not meet above criteria (i.e., those with 0.3 < *ρ* < 0.4 or 0.6 < CI < 0.65).

### 2.6. Gene-Set Enrichment Analysis

We performed gene-set enrichment analysis using the over-representation analysis implemented in the clusterProfiler R package [44]. Gene-sets from the KEGG and the GO biological processes sub-collections of the molecular signatures database (MSigDB v7.2) were used [45,46].

### 2.7. In Vivo Experiment

PDX-1432C (established from a drug naïve TNBC non-BRCA-1 mutated tumor) [47], PDX-0066 (established from a malignant pleural effusion from a patient with BRCA-1 mutated breast cancer) [48], PDX-226 (established from a drug-naïve HER-2 amplified breast cancer tumor) [49], and PDX-434 (established from a drug naïve TNBC BRCA-1 wild type tumor) were generated by the injection of 100,000 cancer cells into the right mammary fat pad of NSG mice, 4–6 mice per group. Control mice were treated with saline, i.p., and the treatment group was treated with 6 mg/kg of cisplatin, i.p., twice with 21 days between the two doses. Mannitol (50 mg/mL) was injected i.p. into each mouse prior to cisplatin chemotherapy to minimize the risk of renal toxicity. The treatment started when the tumors reached 200 mm^3^; tumor growth was monitored with calipers twice per week. All procedures in animals were conducted in accordance with the National Health and Medical Research Council guidelines under the approval of the Austin Animal Ethics Committee. The use of patient samples was approved by Austin Health Human Research Ethics Committee.

Normalized tumor response (NTR) was calculated as the ratio of the tumor volume at the time of the first injection to the smallest tumor volume after the injection (at any time during the experiment), for each mouse. 

In vivo sensitivity of PDXs T250 (BRCA-1 mutated), T127, and T162 (both BRCA-1 methylated) was described in Brugge et al. [37]. Tumor response was assessed based on the proportion of PDXs that resisted the treatment, as determined in [37]. 

### 2.8. Single-Cell Suspension Preparation

The PDX tumors were manually chopped into small pieces (about 1 mm by 1 mm) and resuspended in 10 mL of digestion medium: collagenase IA (300 U/mL) (#C9891, Sigma-Aldrich, St. Louis, MO, USA), hyaluronidase (100 U/mL) (#H3506, Sigma-Aldrich, St. Louis, MO, USA), and deoxyribonuclease I (DNase I) (100 U/mL) (#LS002139, Worthington) in DMEM F12 (#10565018, Thermo Fisher Scientific, Waltham, MA, USA). Samples were incubated for 45 min at 37 °C with agitation and then filtered through a 70 µm cell strainer and spun down for 5 min at 500 g.

### 2.9. mRNA Extraction and Bulk RNA-Seq

For the transcriptomic analysis of PDX tumors, cancer cells were enriched using the Miltenyi mouse cell depletion kit (#130-104-694, Miltenyi Biotec, Bergisch Gladbach, Germany) according to the manufacturer’s recommendations. The mRNA was extracted from the cancer cells using the miRNEasy kit (#217084, Qiagen, Hilden, Germany). Briefly, the mRNA isolation is based on a guanidinium thiocyanate-phenol-chloroform extraction approach. The mRNA is isolated by binding to an exchange column and the genomic DNA is digested on a column by the RNase-Free DNase (#79254, Qiagen, Hilden, Germany). The RNA was finally washed and eluted in water. Quality controls were performed using TapeStation (4200 TapeStation System, Agilent Technologies, Santa Clara, CA, USA), and 100 ng of mRNA was used as input for the library preparation using the TruSeq RNA Library Prep Kit (Illumina, San Diego, CA, USA) according to the manufacturer’s recommendations. The sequencing was performed on a NextSeq 500 instrument using the v2 150 cycle high output kit (Illumina, San Diego, CA, USA). The base calling and quality scoring were determined using Real-Time Analysis on board software v2.4.6 (Illumina, San Diego, CA, USA), while the FASTQ file generation and demultiplexing used bcl2fastq conversion software v2.15.0.4 (Illumina, San Diego, CA, USA).

For the analysis, the RSubread (v2.10.0) and edgeR (v3.38.0) R/Bioconductor packages [39,40] were used to align reads (against human genome hg19), calculate gene counts (using featureCounts), and perform quality control (e.g., using MDS and PCA plots). Technical replicates were assessed and merged for each sample using the sumTechRep function in the edgeR package. Counts were transformed into logRPKM values prior to scoring using singscore [41,42]. Processed RNAseq data for patient samples and matched PDXs was provided by the Jonkers laboratory in the form of count matrices that were then normalized for library size and gene-length biases, and log-transformed to produce logRPKM values.

## 3. Results

### 3.1. Generation of Drug Efficacy Signatures with Training Data Sets

In order to derive new signatures of drug efficacy based on transcriptomic information, we exploited extensive collections of molecular profiling and drug response data generated in cell line screens (i.e., training data [20], see Table 1 for details). Specific gene expression signatures were identified for 90 drugs using RNA sequencing and matching drug response to identify genes whose expression is correlated with sensitivity (as illustrated in Figure S1 for cisplatin). This enabled us to associate each drug with its own transcriptional response signature as shown in Table S1, with the number of genes in these signatures varying between 23 and 253. 

Next, we used singscore [28] to convert gene expression data into signature scores that capture the concordance between the expression profile of an individual sample and the signature associated with a given molecular phenotype (in this case, drug sensitivity). In general, the drug efficacy signature scores correlated highly with those in the training data (*ρ* = 0.7 to *ρ* = 0.86, Figure S2), indicating that the signature score generated by singscore preserved the associations observed between the expression of individual genes and drug response of the corresponding cell line. 

**Figure 1:**
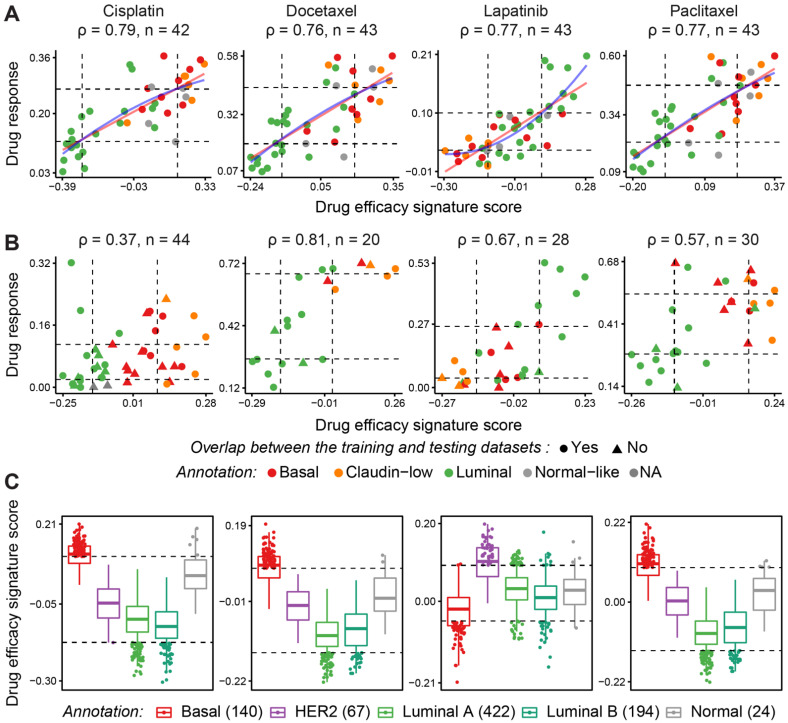
Consistent patterns obtained from drug efficacy signatures across training and test sets for standard of care. Associations between drug efficacy signature scores and drug response (AUC) for four selected drugs in the training data (in (**A**), Gray data), and test sets (in (**B**), from left to right: CCLE for lapatinib, CTRPv2 for docetaxel and paclitaxel, and GDSC1000 for cisplatin). In panel (**C**), TCGA breast cancer samples were scored against these four drug efficacy signatures and stratified by subtypes. Dashed lines in (**A**,**B**) represent the first and third quartiles while in (**C**), they separate the jittered samples with 10%-tile and 90%-tile drug efficacy signature scores. Note that in each of the test sets in B, cell lines are represented with different shapes (triangle and circle) according to their overlap status with the training set.

We then used drug efficacy signature scores as features to build a series of prediction models based on linear and non-linear regression (Figure 1a) and support-vector machines [50] (Methods section *Training prediction models*). Using a cross-validation strategy in the training data, we generated a range of metrics to evaluate model performance, including the root mean squared error (RMSE) which quantifies the difference between a predicted value and the observed value (Figure S3). Based on the cross-validation results, simple linear regression models performed well for most drugs. Linear regression-based classifiers for 87 (out of 90) drugs achieved RMSE under 0.1, while gemcitabine, docetaxel, and paclitaxel showed higher errors across all models (Figure S3). 

### 3.2. Assessing Drug Similarity Based on Observed Response and Prediction

**Figure 2:**
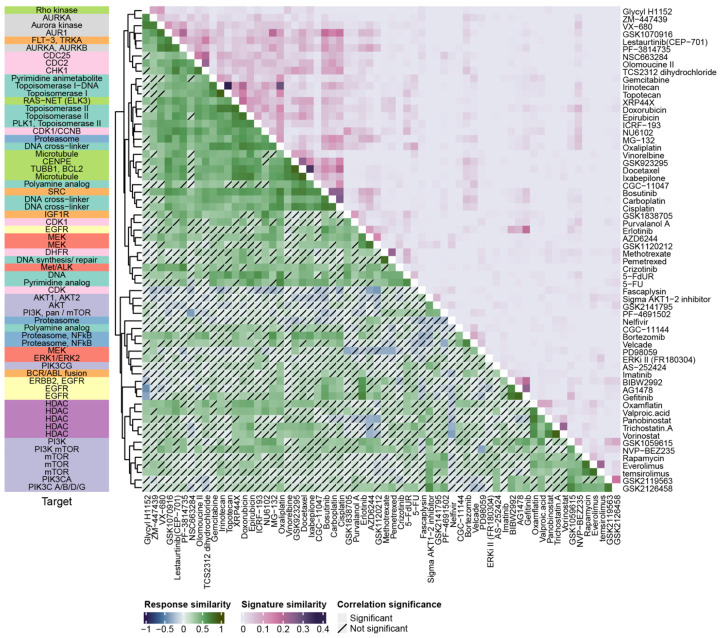
Drug response similarity is retained in drug efficacy signatures. Drug response similarities in the GRAY dataset measured using the Spearman correlation coefficient are shown on the lower triangle of the plot with non-significant correlations (*p*-value > 0.05) crossed out. The upper triangle of the plot represents signature similarities computed using the Jaccard index. Drug classes are labeled on the *y*-axis of the heatmap.

Drugs with similar targets and mechanisms of action will likely elicit similar cellular responses across different cell lines. Since similarities in response represent common molecular mechanisms, these similarities should be captured by response prediction signatures based on molecular measurements. To test this hypothesis, we first computed drug response similarity across the training dataset [9] by computing the Spearman correlation coefficient between the drug efficacies (as measured by area under the dose-response curve, or AUC, obtained from the PharmacoGX R package (v1.6.1). Similarities between the drug response signatures derived in this study were computed using the Jaccard Index, which is often used to measure the degree of set overlap. To better characterize the similarity between responses to different drugs, we annotated drugs with their targets using data from Daemen et al. [20]. As expected, drugs with similar molecular targets elicited similar responses (Figure 2), as demonstrated by the significant similarities within categories such as histone deacetylase (HDAC) targeting drugs and the epidermal growth factor receptor (EGFR) targeting drugs. This similarity was also captured in the response signatures that we developed, thus confirming that our models were retaining molecular information pertaining to the mechanisms of action of each drug.

We next examined whether any gene signaling pathways could be identified in these signatures using GO (Table S3) and KEGG (Table S4) analysis. We saw little enrichment for signaling pathways or processes in the gene sets that we have derived, indicating that these gene sets are capturing information orthogonal to standard pathways and processes. Across all the drug signatures, the most substantial association detected was between the Topotecan, Irinotecan, Nutlin-3, and 5-FU signatures and processes associated with eukaryotic translation, co-translational translocation, and membrane protein localization (Table S3). We also found an association between processes related to wound healing and the GSK1120212 and AZD6244 signatures, and a modest association between the KEGG focal adhesion and small cell lung cancer pathways and our cisplatin signature (Table S4). Otherwise, no substantial pathway enrichment was observed with our gene expression signatures.

### 3.3. Computational Validation Using Independent Testing Datasets

While cross-validation in the training data is an accepted strategy for computational validation, we sought to evaluate the performance of our predictors in the context of independent drug screening data to establish generalizability. Our predictive models were therefore tested in several independent datasets including the CCLE [4], GDSC1000 [29], CTRPv2 [30], gCSI [31,32], and FIMM [33] cell line datasets, as well as on the PDTX-PDTC data (patient-derived tumor xenografts along with PDTX-derived tumor cells) [34] as shown in Table 1. We evaluated our models across all breast cancer data in these independent testing datasets by calculating Spearman’s correlation, *ρ*, the concordance index (CI), RMSE, and mean adjusted error, all of which measure agreement between predicted and observed drug responses. Although many drug efficacy signature scores were highly correlated with drug response in the test datasets, when using the model to predict the area under the dose-response curve (AUC) as a measure of drug sensitivity, we noted that many of the intercepts of the prediction lines shifted. This demonstrates that while the scores accurately order samples from most sensitive to least sensitive, differences in the magnitude of drug response are evident between datasets. This finding agrees with previous observations of reproducibility issues between independent drug response datasets [51], likely due to variations in experimental conditions and sources of cell lines. However, our data confirm the ability of our signatures to distinguish sensitive from resistant samples. Therefore, we considered Spearman’s *ρ* and CI measurements calculated between the predicted and actual drug response to be more suitable metrics for assessing the relationship between our predictions and actual drug response (Figure 1b). Consistent with our observations on the training data (Gray data; Figure S3), a linear regression model performs as well as or better than the other non-linear models in most cases, so we adopt these models for the following analysis.

Of the 90 drugs for which we constructed predictive response signatures, 43 were present in both training and at least one of the testing sets. Across these 43 drugs, our gene expression-based linear prediction models accurately ordered samples from sensitive to resistant for 28, as measured by Spearman’s rank correlation. The other 47 drugs in the training dataset were not present in any of the independent drug screening sets, preventing us from validating their efficacy. We then grouped the 43 drugs with test data available into high, medium, and low confidence according to their *ρ* and CI (Figure S4). Table 2 lists 28 drugs for which we developed models of high and medium confidence, i.e., Spearman’s correlation coefficients were *ρ* ≥ 0.4 (high) or between 0.4 and 0.3 (medium) between drug efficacy signature scores and observed drug response in at least one independent testing set (see Table S2 for *ρ* of all drugs). For some drugs such as lapatinib, the efficacy scores computed on the Caldas PDTX data had a strong correlation of 0.9 with the observed drug response, highlighting the fact that our signatures were consistent across different biological models.

Some of the drugs which showed high and medium confidence in our predictive models are currently used as a standard of care in the clinic (i.e., docetaxel, doxorubicin, lapatinib, and paclitaxel), and are known to show differences in efficacy across different molecular subtypes of breast cancer. To investigate whether our signatures can predict these differences, we studied the associations between drug efficacy signature scores and drug response in each breast cancer subtype for these four drugs (Figure 1). As expected, cisplatin, docetaxel, and paclitaxel were predicted to have greater efficacy in triple-negative breast cancer (TNBC), and lapatinib in the HER-2 amplified subtype, using cell lines from the training (Figure 1a) or validation (Figure 1b) datasets. We further showed that these subtype-dependent patterns were consistent in TCGA data from breast cancer patients, where samples with the top and bottom 10% of drug efficacy scores showed subtype specificity in response to cisplatin, docetaxel, lapatinib, and paclitaxel (Figure 1c). Overall, these results demonstrate that gene expression signatures generated from a limited set of cell lines were predictive of response in other cell lines for at least half of the drugs that we examined and that the patterns of drug scores obtained from the drug efficacy signatures were highly conserved across cell lines and patient data.

### 3.4. Validation of Response Predictions in Patient-Derived Xenografts

**Figure 3:**
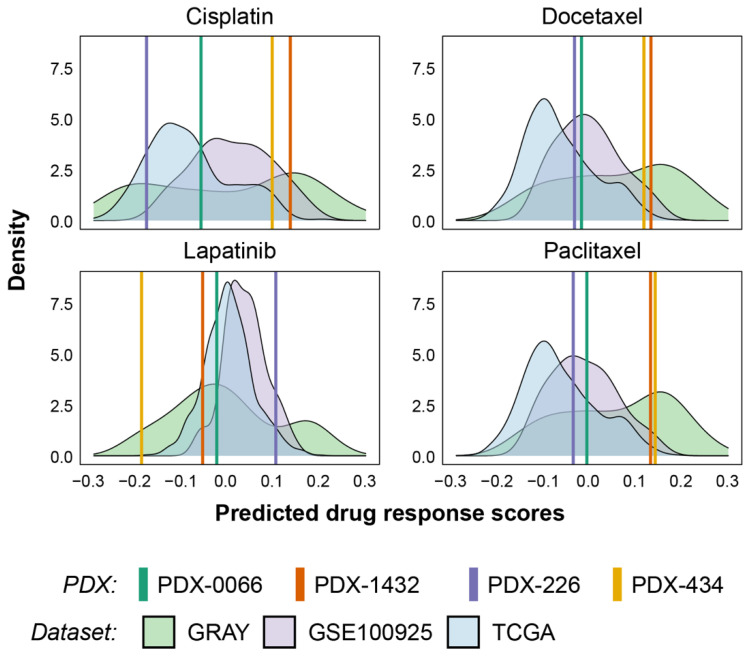
Dynamic range of predicted response in PDX tumors is comparable to that from publicly available datasets. Drug efficacy scores computed for the PDX tumors and publicly available patient and cell line datasets using stingscore, exhibit a wide range of predicted responses. Bimodal distributions are noticeable in most plots, highlighting the presence of responsive and resistant populations within each dataset.

**Figure 4:**
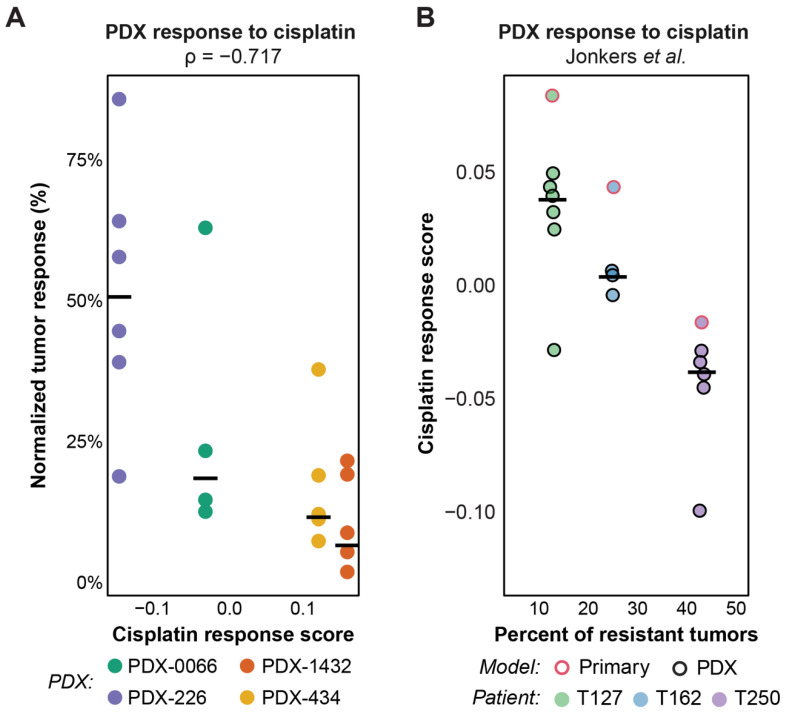
Cisplatin efficacy scores in pre-clinical models accurately discriminate between responsive and resistant tumors. (**A**) Cisplatin efficacy scores for individual mice bearing one of four PDX tumors are anti-correlated with median estimates of NTR (normalized tumor response), estimated from growth curves (Figure S5). (**B**) Cisplatin efficacy scores discriminate between BRCA1-deficient PDX models based on their likelihood to respond to the drug. Efficacy scores are inversely correlated with the proportion of resistant tumors (defined in 47) and thus are predictive of resistance in BRCA1-deficient models.

Having validated our computational models on publicly available cell line and PDTX datasets, we sought to further validate the drugs described in Figure 1 using TNBC patient-derived xenografts (PDXs). We first assessed the range of responses predicted using our drug response prediction signatures for cisplatin, docetaxel, lapatinib, and paclitaxel (Figure 3). Data from TCGA, CCLE, and GSE100925 were used to compute response prediction scores across breast cancer patients and cell lines. To enable cross-dataset comparisons, we used the stingscore method to compute drug efficacy scores as the method was designed to correct for dataset biases using the expression of endogenous “control” genes [42]. A range of responses was predicted across all three datasets as shown in Figure 3, with most predicted responses showing a multi-modal distribution that was suggestive of responsive and resistant populations. We then overlayed scores computed from the transcriptomic analysis of four TNBC PDX models for each of the four drugs and showed a similar dynamic range of predicted response values. Surprisingly, despite the recognized greater efficacy of platinum therapies on BRCA-1 mutated tumors [15], the two PDXs with the higher prediction scores for cisplatin in this cohort were not BRCA-1 mutated (PDX-434 and PDX-1432C). We then assessed their sensitivity to cisplatin in vivo and confirmed that PDX-434 and PDX-1432C remained highly responsive (Figure S5). As an indicator of response, we used a normalized tumor response (NTR) value, calculated as the ratio of the tumor volume at the time of the first drug injection to the smallest tumor volume measured after the injection. We confirmed that cisplatin response prediction scores were highly anti-correlated with NTR values (Figure 4a), suggesting the robust nature of our gene expression-based drug response prediction models. Furthermore, this result highlighted that our predictive signature for cisplatin, which is based on transcriptomic profiling rather than mutational analysis, enabled us to identify rare cases of breast cancer patients who are likely to respond to cisplatin, despite not being identified as belonging to the ‘BRCA-ness’ subgroup based on routine genomic testing.

To demonstrate whether this signature could predict the sensitivity of BRCA-1 deficient (mutated or hypermethylated) tumors, we used a publicly available dataset from a collection of BRCA-1 deficient PDXs [37]. Based on the signature validated with our in-house PDXs (Figure 4a), we then predicted the cisplatin response across the BRCA-1 deficient PDXs (Figure 4b) and found that our predictions correlated with the overall proportion of resistant tumors when treated with cisplatin in vivo (Figure 4b). Interestingly, the primary tissues from all three corresponding patients had a slightly higher predicted sensitivity score compared to their respective PDXs. It would be interesting to determine whether a particular clonal selection occurred in vivo or whether the influence of the mouse host tumor microenvironment was responsible for the difference in cisplatin sensitivity. Furthermore, some variations were observed between mice of the same model (Figure 4a,b), likely due to inter-tumoral heterogeneity. It would be interesting to score each tumor independently to determine whether sensitivity to cisplatin can be assessed at the individual level.

Altogether, these results indicate that gene expression profiling can be used to predict response to cisplatin, regardless of the BRCA-1 status or deficiency of the tumors. This highlights the substantial information that can be gained from transcriptomic analysis as a guide to therapy selection.

## 4. Discussion

In this study, we developed and validated, in silico, a set of robust computational models to predict drug responses for 90 compounds. While it has been demonstrated previously that methods using gene expression correlated with drug response are predictive of sensitivity and resistance (e.g., [52,53,54]), we used this approach in conjunction with our recently developed single-sample scoring methods, singscore and stingscore, to generate per-sample drug efficacy scores. These methods provide a simple and intuitive way of converting gene expression profiles into numeric values for classification and have been used previously in the classification of molecular phenotypes [42,55,56], prediction of mutation status [57], and prediction of tumor-infiltrating lymphocytes in melanoma and colorectal cancer [58,59].

While some research groups have attempted to obtain predictive features that are shared across multiple cancer types by analyzing pan-cancer cohorts [60], cancer-specific classifiers are more likely to account for tissue-specific features [61]. Here, we applied our panel of drug efficacy expression signatures across multiple independent breast cancer cell lines and patient sample datasets. To develop an unbiased approach and predict efficacy regardless of the molecular subtype, we did not stratify cell lines according to their molecular subtypes in our training data. Despite this, our drug efficacy signature scores captured known subtype-specific differences in response for drugs, such as cisplatin, docetaxel, and paclitaxel, which have been shown to be more effective in TNBC, and lapatinib which is known to be effective for treating HER2-positive tumors. However, for most drugs (e.g., panobinostat, vorinostat, and doxorubicin), molecular subtypes do not explain the observed differences in treatment response. For these drugs, our scoring approach may provide an avenue to guide the selection of targeted therapeutics, regardless of their molecular subtype or genomic status.

We noted relatively little overlap between our response signatures and previously published gene expression signatures associated with prognosis [54,62,63], with the only notable overlap between our cisplatin response signature and a signature associated with response to neoadjuvant chemotherapy in breast cancer [54], which shared 6 genes out of 192.

Our in silico scoring strategy confirmed differences in sensitivity to the same treatment in patients sharing the same disease subtype. Thus, our model could be used not only to explore the biology that underlies the heterogeneous drug responses but also to provide a useful guide to prioritizing drugs for patients within a given subtype. Importantly, we found that our prediction signature for cisplatin could predict sensitivity to the drug within the TNBC subtype, regardless of their BRCA-1 mutation status, and we validated this in silico observation in PDX models. While other mutations in genes from the DNA repair pathway could also be predictive of cisplatin response in these models, our results indicate that transcriptomic data contains valuable information to identify drug responders. This is of high clinical significance because clinical trials have previously shown that a small proportion of BRCA-1 wild-type breast cancer patients can benefit from platinum-based therapies [15]. It would be interesting to determine whether this signature for cisplatin could be used to predict the sensitivity of testicular gem cell and ovarian cancers, which are known to be sensitive to this drug [64]. However, the current lack of training and validation data makes cross-cancer application and testing difficult.

As baseline transcriptional data were used to generate and validate these signatures, we anticipate that this strategy could be used to predict sensitivity prior to any treatment. Experiments using time-course analysis would be useful to determine whether some of these genes are deregulated in response to drug exposure, or if the treatment can select the emergence of resistant clones within the initial population. Our predictions are based on bulk RNA sequencing and, therefore, are likely to be predictive of the responses of dominant clones present in the biopsied lesions. As a consequence, drugs associated with positive predictions may have a drastic effect on primary tumor or metastatic burden, improving the health of the patients. While the optimization of this approach at the single cell level may enable the prediction of efficacy of both minor and dominant clones, the use of single-cell sequencing in personalized medicine is still challenging due to the number of cells and genes per cell that can be analyzed for each patient, as well as the cost associated with this kind of analysis. Our results indicate that scoring of bulk RNA sequencing data might be a good indicator of clinical response for the lesions that are biopsied, and that multiple biopsies may be required to tailor individual patient therapies over time. Likewise, biopsies from multiple sites will also help to capture heterogeneity and identify likely variations in drug sensitivity due to clonal differences in metastases. Sampling this variation, in combination with predictions of drug efficacy, presents an opportunity to personalize therapy.

While gene expression data can successfully predict drug response for many drugs, it is likely that for other drugs, the responsiveness will be better explained by specific mutations, genomic or epigenomic changes, or post-transcriptomic events that regulate protein function. For example, over 30 years ago, mutations in ESR1 were reported for the first time to be associated with resistance to hormone therapies in ER-positive cancers [65] and more recent evidence confirms this observation [66]. Recupero et al. showed that truncation of HER2 in breast cancer may cause resistance to trastuzumab [67]. Thus, combining gene expression with other molecular information, such as the mutation of cancer driver genes or drug resistance genes, may improve the performance of predictions for some drugs. In the case of cisplatin, BRCA-1 expression levels and methylation of the BRCA-1 promotor are also important in mediating sensitivity [37,68]. Furthermore, a recent study identified genomes associated with cisplatin resistance at a clonal level [69]. While the predictive signature we present for cisplatin does not itself contain BRCA-1 or -2, it would be interesting to extend predictive models to take several -omics analyses into consideration and evaluate the fitness of resistant clones under treatment pressure [69,70].

Our findings demonstrate that accurate prediction of drug response based on gene expression features holds great hope for optimizing and personalizing treatment for cancer patients, and approaches such as the one we have developed here will continue to gain power as datasets improve.

## 5. Conclusions

Overall, our study demonstrates that drug prediction based on transcriptomic profiling can be applied to any sample and contains information that cannot be identified by mutation status, for instance in the case of cisplatin. Combining this powerful and general approach with the identification of more sparse, and therefore less frequently detected, actionable mutations will enable the further personalization of treatments in the future.

## Figures and Tables

**Table 1:** Training and test datasets used to derive and test the drug efficacy signatures TN: triple-negative; AUC: area under the dose-response curve; RCB: residual cancer burden; PDTX-PDTC: patient-derived tumor xenografts and PDTX-derived tumor cells; NTR: Normalized tumor response. Note that *N_Samples_* represents the total number of cell lines/tissue samples in each study, however, not all of these were used for drug screening and some cell lines did not have both RNA-seq and drug data.

Data Set	*N_Samples_*	Type of Sample	*N_Drugs_*	RNASeq	Microarray	Response Metrics	Use	Ref
Gray	84	Cell line	90	Yes	Yes	AUC	Train	[20]
CCLE	60	Cell line	24	Yes	Yes	AUC	Test	[4]
GDSC1000	50	Cell line	251	No	Yes	AUC	Test	[29]
CTRPv2	40	Cell line	545	From CCLE	No	AUC	Test	[30]
gCSI	30	Cell line	16	Yes	No	AUC	Test	[31,32]
FIMM	21	Cell line	52	From CCLE	No	AUC		[33]
Caldas	20	PDTX-PDTC	104	No	Yes	AUC	Test	[34]
TCGA	1102	Patient	-	Yes	Yes	-	Test	GSE62944 [35]
GSE100925	50	Patient	-	Yes	-	-	Test	GSE100925
GSE103668 (cisplatin)	21 TN	Clinical trial	1	No	Yes	Miller-Payne and RCB	Test	[36]
ONJCRI-PDX	4	PDX	1	Yes	No	NTR	Test	In-house
Jonkers-PDX	3	PDX	1	Yes	No	Proportion of remissions and resistance	Test	[37]

**Table 2:** Spearman’s correlation coefficients (*ρ*) between drug efficacy signature scores and observed drug response. The Gray data were used to generate drug efficacy signature scores. Drugs with *ρ* ≥ 0.4 in at least one test set (high-confidence) and drugs with 0.4 > *ρ* ≥ 0.3 (medium confidence) are shown. Table S2 shows this information for all the 90 drugs.

Drugs	GRAY	CCLE	CTRPv2	FIMM	gCSI	GDSC1000	Caldas	Confidence
AZD6244	0.72	0.42	0.42	0.28	-	-	-	High
Bortezomib	0.68	-	0.29	0.43	0.4	0.41	0.5	High
Docetaxel	0.76	-	0.8	-	0.48	0.43	0.43	High
Doxorubicin	0.78	-	-	0.28	0.6	−0.05	-	High
Erlotinib	0.76	0.41	0.31	−0.09	0.57	0.3	−0.13	High
Gefitinib	0.74	-	0.37	0.22	-	0.33	0.47	High
Gemcitabine	0.75	-	0.35	-	0.48	0.19	0.3	High
GSK1059615	0.73	-	0.49	-	-	-	-	High
GSK1120212	0.78	-	0.54	-	-	-	0.19	High
GSK461364	0.78	-	0.72	-	-	-	-	High
Irinotecan	0.8	0.13	-	−0.13	0.57	-	-	High
Lapatinib	0.77	0.68	0.54	0.5	0.34	0.26	0.9	High
MG-132	0.76	-	0.47	-	-	0.31	-	High
Nutlin-3	0.74	0.22	0.42	-	-	-	-	High
Paclitaxel	0.77	0.36	0.61	0.37	0.42	0.12	−0.1	High
Panobinostat	0.78	0.76	0.6	0.72	-	-	-	High
Rapamycin	0.7	-	0.44	-	−0.17	−0.05	-	High
Topotecan	0.76	0.6	0.36	0.15	-	-	-	High
VX-680	0.69	-	0.52	-	-	0.21	-	High
ZM-447439	0.7	-	-	-	-	0.28	0.46	High
5-FU	0.77	-	0.31	-	-	-	-	Medium
BIBW2992	0.79	-	0.4	0.39	-	-	0.37	Medium
Cisplatin	0.79	-	-	-	-	0.37	0.29	Medium
Crizotinib	0.75	0.21	0.36	−0.04	0.39	0.03	-	Medium
Etoposide	0.75	-	0.38	-	-	0.21	-	Medium
GSK2126458	0.73	-	-	-	-	0.33	-	Medium
Methotrexate	0.74	-	0.18	0.09	-	0.39	-	Medium
Temsirolimus	0.72	-	-	0.4	-	0.1	-	Medium

## Data Availability

RNA sequencing data can be made available upon request.

## Supplementary Materials

The following supporting information can be downloaded at: https://www.mdpi.com/article/10.3390/cancers14102404/s1. Supplementary Figure S1 Heatmap illustrating z-transformed (within each gene) expression of the cisplatin signature genes in the training data. Supplementary Figure S2. Associations between the drug efficacy signature scores and the observed drug response (area under the dose-response curve). Supplementary Figure S3. Assessment of model performance. Supplementary Figure S4. Performance of the prediction models (linear regression) in the test data sets. Supplementary Figure S5. Growth curves of 4 TNBC PDXs treated in vivo with cisplatin. Supplementary Table S1: Drug efficacy signatures derived in this study. The direction of each gene determines its correlation with drug efficacy, ‘Up’ represents correlated genes and ‘Down’ represents anti-correlated genes. Supplementary Table S2: Spearman’s correlation coefficients between drug efficacy signature scores and observed drug response for all drugs. Supplementary Table S3: List of GO pathways identified for each drug response signature. Supplementary Table S4: List of the KEGG pathways identified for each drug response signature.
