# Supplementary material for: Computational Screening of Anti-Cancer Drugs Identifies a New BRCA Independent Gene Expression Signature to Predict Breast Cancer Sensitivity to Cisplatin
Source: Cancers (Basel). 2022 May 13;14(10):2404. doi: 10.3390/cancers14102404 (PMC9139442; doi:10.3390/cancers14102404)
Supplement: Supplementary file 1 [file cancers-14-02404-s001.zip › cancers-1699456 sups figs.pdf]

Supplementary results

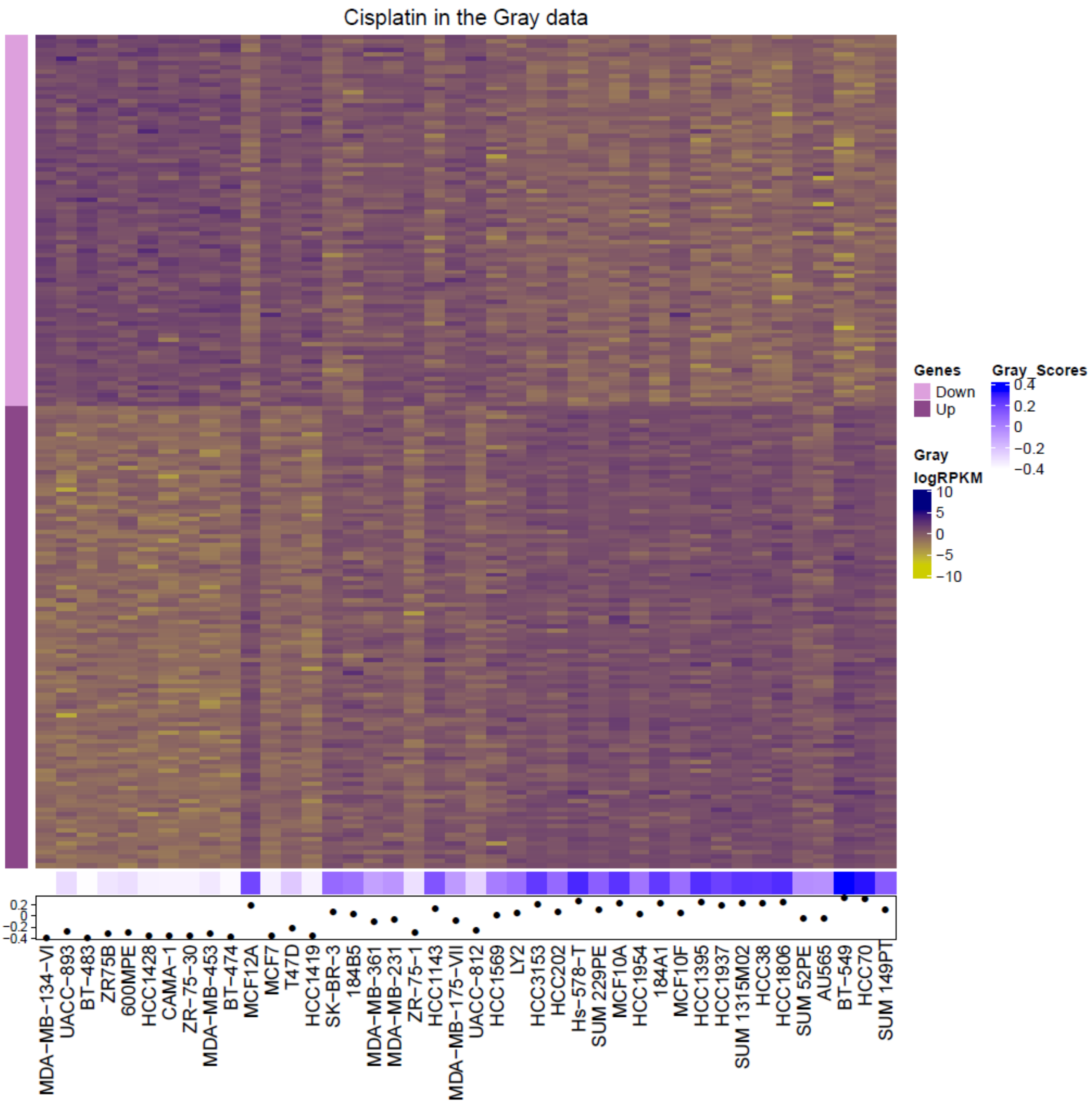

Figure S1. Heatmap illustrating z-transformed (within each gene) expression of the cisplatin signature genes in the training data (left) and patient data (right). In the left heatmap, each row corresponds to a gene and columns contain cell lines (from Gray data) that are sorted by drug sensitivity (AUC) from the least sensitive cell line (left) to most sensitive (right). "Up" genes are genes that are positively correlated with drug response, and "Down" represents genes that are negatively correlated with drug response.

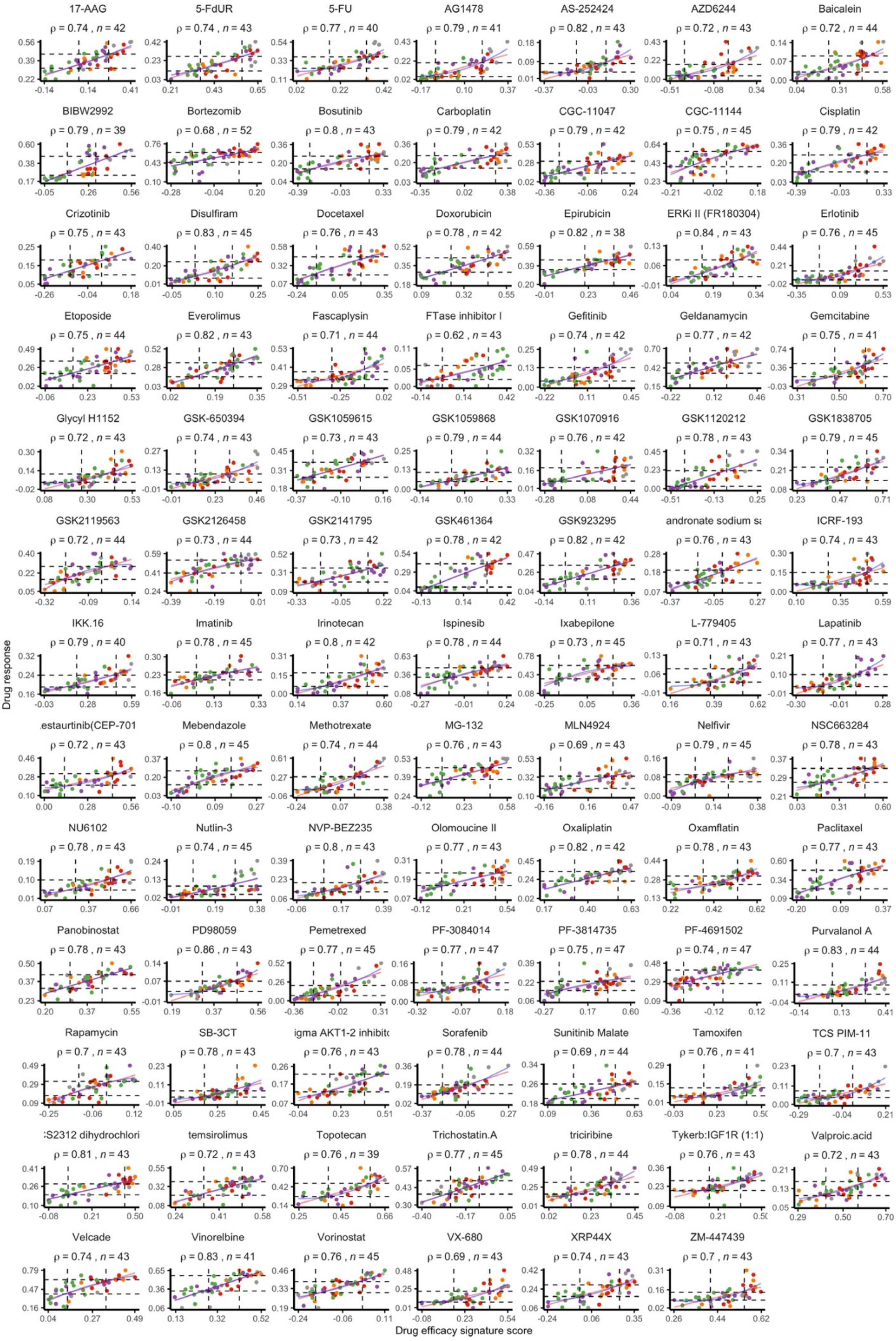
17-AAG
ρ = 0.74 , n = 42
5-FdUR
ρ = 0.74 , n = 43
5-FU
ρ = 0.77 , n = 40
AG1478
ρ = 0.79 , n = 41
AS-252424
ρ = 0.82 , n = 43
AZD6244
ρ = 0.72 , n = 43
Baicalein
ρ = 0.72 , n = 44
BIBW2992
ρ = 0.79 , n = 39
Bortezomib
ρ = 0.68 , n = 52
Bosutinib
ρ = 0.8 , n = 43
Carboplatin
ρ = 0.79 , n = 42
CGC-11047
ρ = 0.79 , n = 42
CGC-11144
ρ = 0.75 , n = 45
Cisplatin
ρ = 0.79 , n = 42
Crizotinib
ρ = 0.75 , n = 43
Disulfiram
ρ = 0.83 , n = 45
Docetaxel
ρ = 0.76 , n = 43
Doxorubicin
ρ = 0.78 , n = 42
Epirubicin
ρ = 0.82 , n = 38
ERKi II (FR180304)
ρ = 0.84 , n = 43
Erlotinib
ρ = 0.76 , n = 45
Etoposide
ρ = 0.75 , n = 44
Everolimus
ρ = 0.82 , n = 43
Fascaplysin
ρ = 0.71 , n = 44
FTase inhibitor I
ρ = 0.62 , n = 43
Gefitinib
ρ = 0.74 , n = 42
Geldanamycin
ρ = 0.77 , n = 42
Gemcitabine
ρ = 0.75 , n = 41
Glycyl H1152
ρ = 0.72 , n = 43
GSK-650394
ρ = 0.74 , n = 43
GSK1059615
ρ = 0.73 , n = 43
GSK1059868
ρ = 0.79 , n = 44
GSK1070916
ρ = 0.76 , n = 42
GSK1120212
ρ = 0.78 , n = 43
GSK1838705
ρ = 0.79 , n = 45
GSK2119563
ρ = 0.72 , n = 44
GSK2126458
ρ = 0.73 , n = 44
GSK2141795
ρ = 0.73 , n = 42
GSK461364
ρ = 0.78 , n = 42
GSK923295
ρ = 0.82 , n = 42
andronate sodium sa
ρ = 0.76 , n = 43
ICRF-193
ρ = 0.74 , n = 43
IKK.16
ρ = 0.79 , n = 40
Imatinib
ρ = 0.78 , n = 45
Irinotecan
ρ = 0.8 , n = 42
Ispinesib
ρ = 0.78 , n = 44
Ixabepilone
ρ = 0.73 , n = 45
L-779405
ρ = 0.71 , n = 43
Lapatinib
ρ = 0.77 , n = 43
.estaurtinib(CEP-701
ρ = 0.72 , n = 43
Mebendazole
ρ = 0.8 , n = 45
Methotrexate
ρ = 0.74 , n = 44
MG-132
ρ = 0.76 , n = 43
MLN4924
ρ = 0.69 , n = 43
Nelfivir
ρ = 0.79 , n = 45
NSC663284
ρ = 0.78 , n = 43
NU6102
ρ = 0.78 , n = 43
Nutlin-3
ρ = 0.74 , n = 45
NVP-BEZ235
ρ = 0.8 , n = 43
Olomoucine II
ρ = 0.77 , n = 43
Oxaliplatin
ρ = 0.82 , n = 42
Oxamflatin
ρ = 0.78 , n = 43
Paclitaxel
ρ = 0.77 , n = 43
Panobinostat
ρ = 0.78 , n = 43
PD98059
ρ = 0.86 , n = 43
Pemetrexed
ρ = 0.77 , n = 45
PF-3084014
ρ = 0.77 , n = 47
PF-3814735
ρ = 0.75 , n = 47
PF-4691502
ρ = 0.74 , n = 47
Purvalanol A
ρ = 0.83 , n = 44
Rapamycin
ρ = 0.7 , n = 43
SB-3CT
ρ = 0.78 , n = 43
igma AKT1-2 inhibit
ρ = 0.76 , n = 43
Sorafenib
ρ = 0.78 , n = 44
Sunitinib Malate
ρ = 0.69 , n = 44
Tamoxifen
ρ = 0.76 , n = 41
TCS PIM-11
ρ = 0.7 , n = 43
:S2312 dihydrochlori
ρ = 0.81 , n = 43
temsirolimus
ρ = 0.72 , n = 43
Topotecan
ρ = 0.76 , n = 39
Trichostatin.A
ρ = 0.77 , n = 45
triciribine
ρ = 0.78 , n = 44
Tykerb:IGF1R (1:1)
ρ = 0.76 , n = 43
Valproic.acid
ρ = 0.72 , n = 43
Velcade
ρ = 0.74 , n = 43
Vinorelbine
ρ = 0.83 , n = 41
Vorinostat
ρ = 0.76 , n = 45
VX-680
ρ = 0.69 , n = 43
XRP44X
ρ = 0.74 , n = 43
ZM-447439
ρ = 0.7 , n = 43
Drug response
Drug efficacy signature score

Figure S2. Associations between the drug efficacy signature scores and the observed drug response (area under the dose-response curve). ρ: Spearman's correlation coefficient, n: number of cell lines; coloured points represent different subtypes; the fitted lines from linear regression (*red*) and quadratic regression (*blue*) are shown. All drug efficacy signatures and the fitted lines were derived from the Gray data.

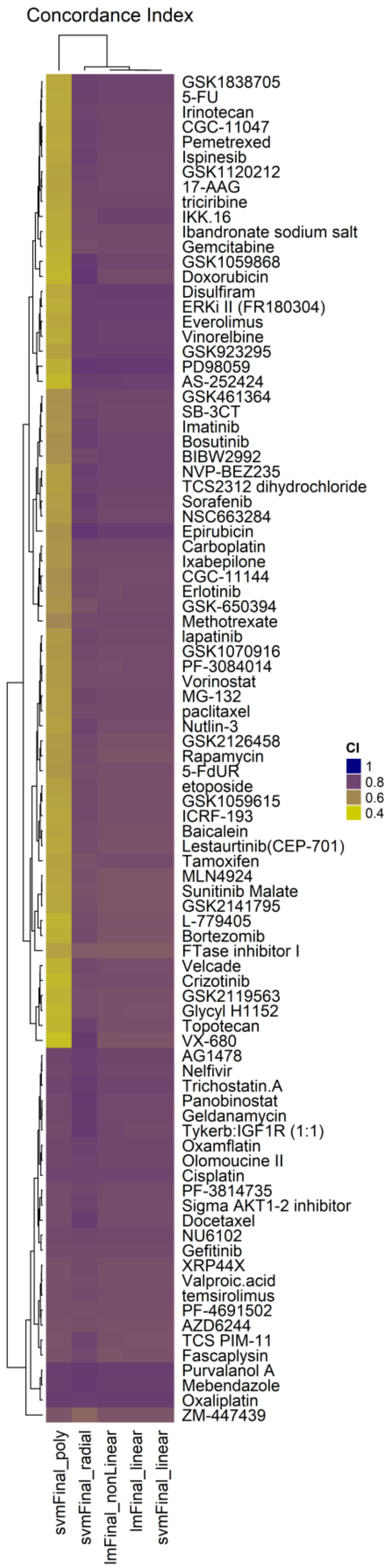
Concordance Index
GSK1838705
5-FU
Irinotecan
CGC-11047
Pemetrexed
Ispinesib
GSK1120212
17-AAG
triciribine
IKK.16
Ibandronate sodium salt
Gemcitabine
GSK1059868
Doxorubicin
Disulfiram
ERKi II (FR180304)
Everolimus
Vinorelbine
GSK923295
PD98059
AS-252424
GSK461364
SB-3CT
Imatinib
Bosutinib
BIBW2992
NVP-BEZ235
TCS2312 dihydrochloride
Sorafenib
NSC663284
Epirubicin
Carboplatin
Ixabepilone
CGC-11144
Erlotinib
GSK-650394
Methotrexate
lapatinib
GSK1070916
PF-3084014
Vorinostat
MG-132
paclitaxel
Nutlin-3
GSK2126458
Rapamycin
5-FdUR
etoposide
GSK1059615
ICRF-193
Baicalein
Lestaurtinib(CEP-701)
Tamoxifen
MLN4924
Sunitinib Malate
GSK2141795
L-779405
Bortezomib
FTase inhibitor I
Velcade
Crizotinib
GSK2119563
Glycyl H1152
Topotecan
VX-680
AG1478
Nelfivir
Trichostatin.A
Panobinostat
Geldanamycin
Tykerb:IGF1R (1:1)
Oxamflatin
Olomoucine II
Cisplatin
PF-3814735
Sigma AKT1-2 inhibitor
Docetaxel
NU6102
Gefitinib
XRP44X
Valproic.acid
temsirolimus
PF-4691502
AZD6244
TCS PIM-11
Fascaplysin
Purvalanol A
Mebendazole
Oxaliplatin
ZM-447439
svmFinal_poly
svmFinal_radial
lmFinal_nonLinear
lmFinal_linear
svmFinal_linear
CI
1
0.8
0.6
0.4

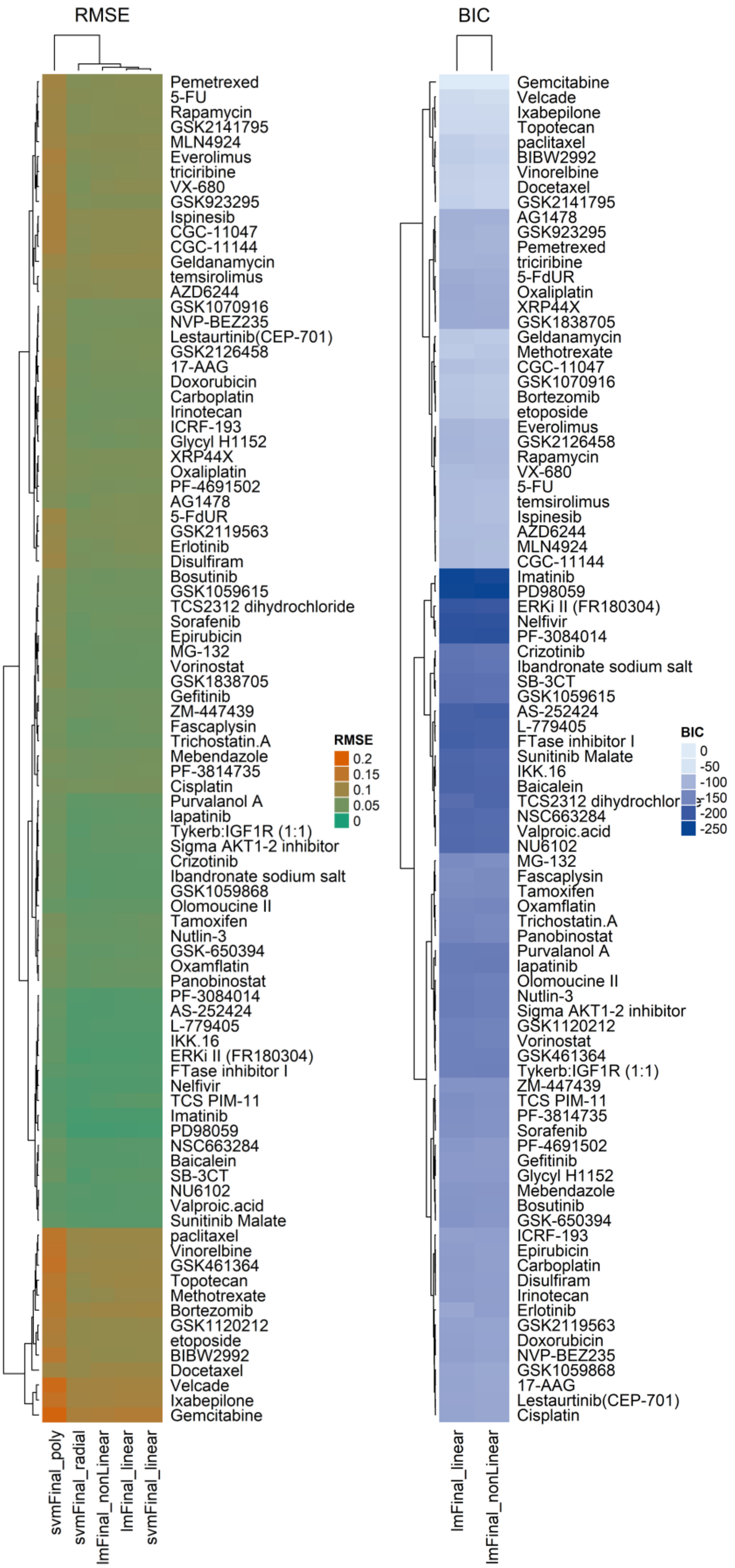

Figure S3. Assessment of model performance. Model performance was evaluated using the concordance index (CI), the root mean square error (RMSE) and the Bayesian information criteria (BIC). Low RMSE, high CI and low BIC are indicative of a well performing model.

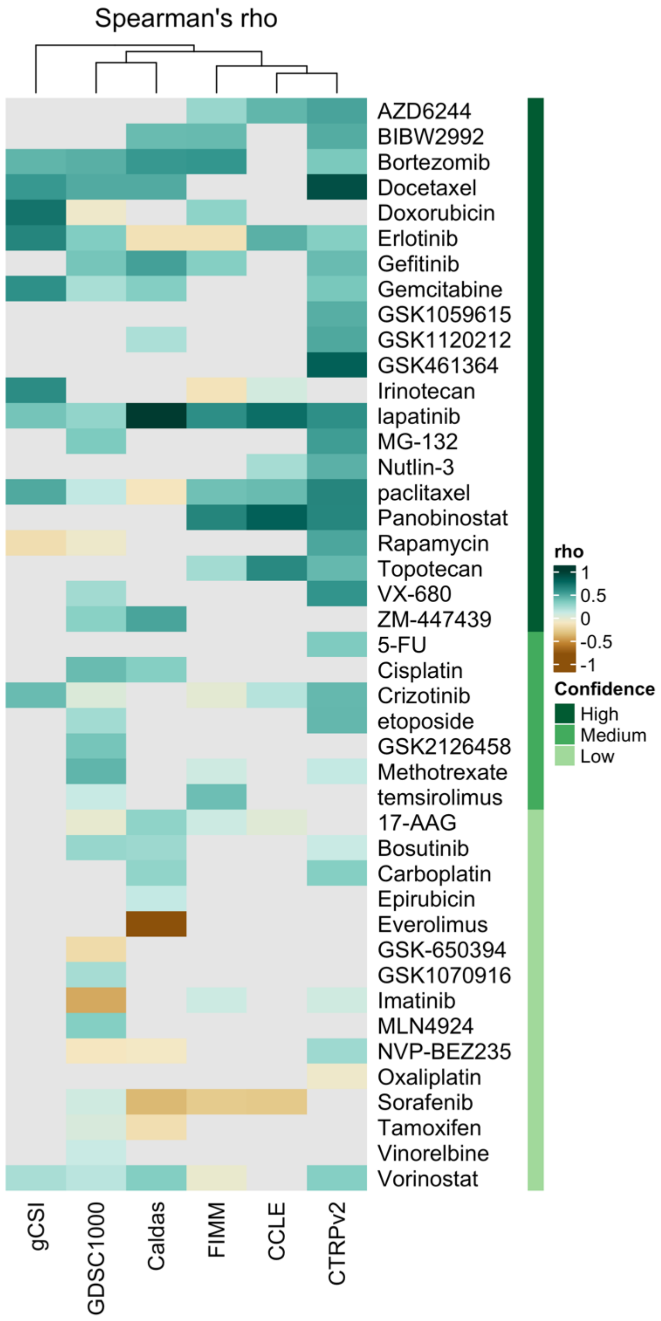
Spearman's rho
AZD6244
BIBW2992
Bortezomib
Docetaxel
Doxorubicin
Erlotinib
Gefitinib
Gemcitabine
GSK1059615
GSK1120212
GSK461364
Irinotecan
lapatinib
MG-132
Nutlin-3
paclitaxel
Panobinostat
Rapamycin
Topotecan
VX-680
ZM-447439
5-FU
Cisplatin
Crizotinib
etoposide
GSK2126458
Methotrexate
temsirolimus
17-AAG
Bosutinib
Carboplatin
Epirubicin
Everolimus
GSK-650394
GSK1070916
Imatinib
MLN4924
NVP-BEZ235
Oxaliplatin
Sorafenib
Tamoxifen
Vinorelbine
Vorinostat
gCSI
GDSC1000
Caldas
FIMM
CCLE
CTRPv2
rho
1
0.5
0
-0.5
-1
Confidence
High
Medium
Low

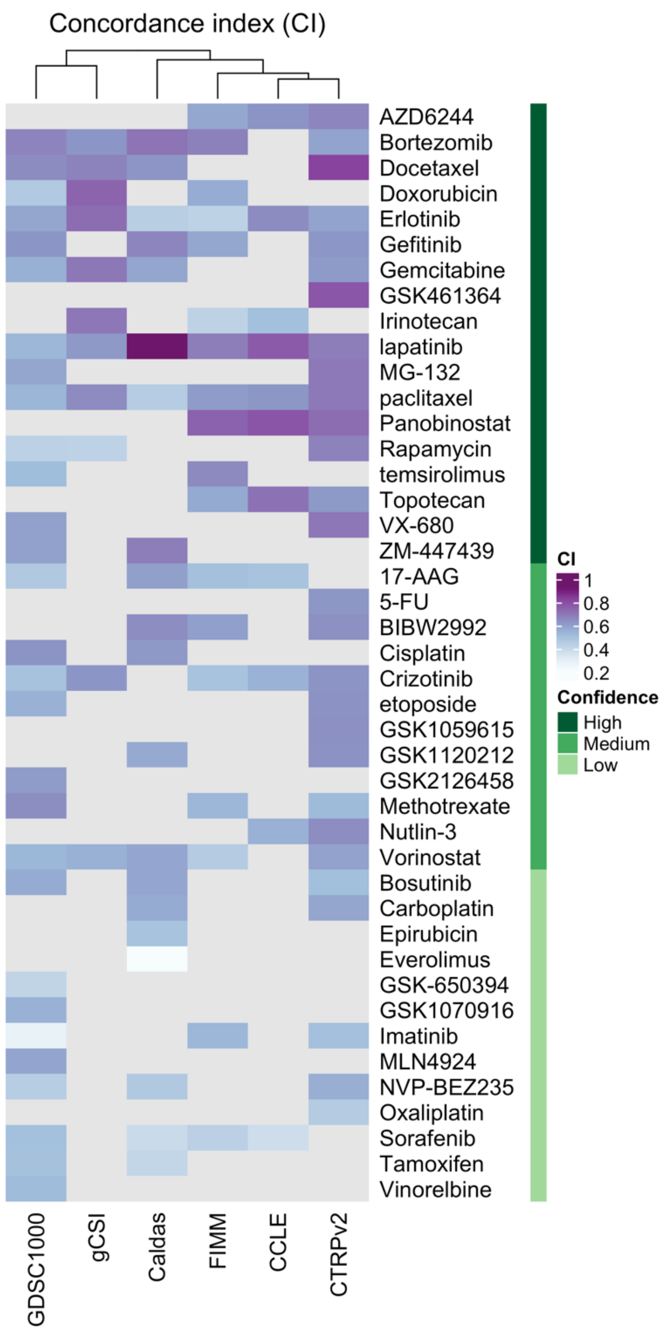

Figure S4. Performance of the prediction models (linear regression) in the test datasets. Spearman's correlation coefficient (left) and the concordance index (right) were computed using test datasets for linear regression models trained on drug efficacy scores. High correlation coefficient and CIs indicate better performance of the model on the test data. Drugs have been annotated based on their performance in the training data with high confidence drugs representing models where performance on training data was good.

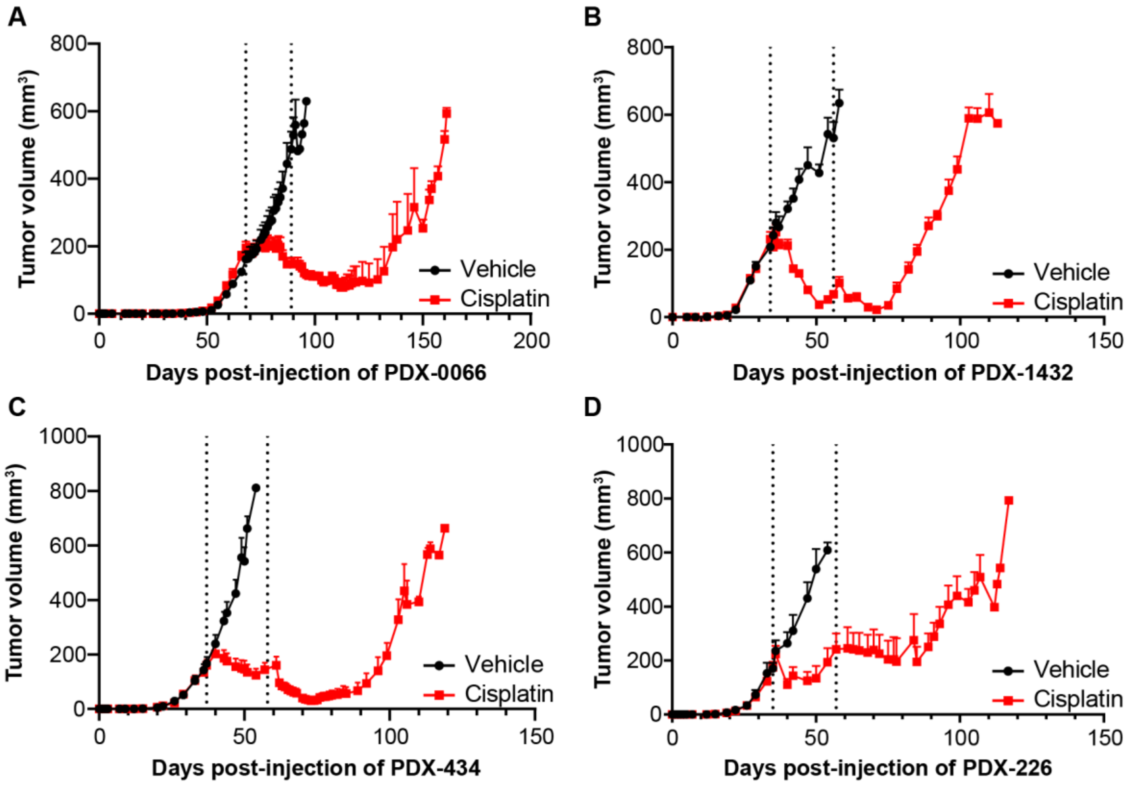

Figure S5. Growth curves of four different PDX tumors treated with cisplatin. Mice bearing PDX-0066 (**A**, 4 mice per group), PDX-1432C (**B**, 6 mice per group), PDX-434 (**C**, 5 mice per group) or PDX-226 (**D**, 6 mice per group) were treated with vehicle (saline, i.p.) or cisplatin, (6 mg/kg, i.p.). The dash lines indicate the times of treatment. The data represent the means +/- S.E.M.
